# Electrochemical disinfection of repeatedly recycled blackwater in a free‐standing, additive‐free toilet

**DOI:** 10.1111/wej.12277

**Published:** 2017-07-23

**Authors:** Brian T. Hawkins, Katelyn L. Sellgren, Ethan J. D. Klem, Jeffrey R. Piascik, Brian R. Stoner

**Affiliations:** ^1^ RTI International Research Triangle Park NC USA; ^2^ Pratt School of Engineering Duke University Durham NC USA

**Keywords:** decentralized waste water treatment, electrochemistry, sustainability, water reuse

## Abstract

Decentralized, energy‐efficient waste water treatment technologies enabling water reuse are needed to sustainably address sanitation needs in water‐ and energy‐scarce environments. Here, we describe the effects of repeated recycling of disinfected blackwater (as flush liquid) on the energy required to achieve full disinfection with an electrochemical process in a prototype toilet system. The recycled liquid rapidly reached a steady state with total solids reliably ranging between 0.50 and 0.65% and conductivity between 20 and 23 mS/cm through many flush cycles over 15 weeks. The increase in accumulated solids was associated with increased energy demand and wide variation in the free chlorine contact time required to achieve complete disinfection. Further studies on the system at steady state revealed that running at higher voltage modestly improves energy efficiency, and established running parameters that reliably achieve disinfection at fixed run times. These results will guide prototype testing in the field.

## Introduction

An estimated 2.5 billion people worldwide lack modern electrical and clean water infrastructures, resulting in millions of deaths annually from diseases acquired through the use of unsafe water resources (Wardlaw *et al*. 2010; Elledge & McClatchey 2013). Distribution of clean water depends upon having the energy available to treat waste water effectively. One approach to quickly address the lack of clean water in lower income countries is to reduce the dependence on energy‐ and infrastructure‐intensive waste water treatment technology. Our research is focused on developing a free‐standing disinfection system for human waste that does not require added water, chemical reagents, or energy in excess of what can be produced at the point of use, is cost‐effective and produces non‐potable water suitable for reuse as flush water.

Previously, we demonstrated that electrochemical disinfection with an off‐the‐shelf mixed metal oxide (MMO) electrochemical cell was effective at disinfecting both model waste water (diluted urine spiked with *Escherichia coli*) and blackwater, consisting of fecal‐contaminated urine and recycled flush water, from our toilet prototype, but that the latter required longer running times and greater energy inputs per liter to achieve adequate disinfection (Sellgren *et al*. 2017). Here, we expand on those observations, and describe: (1) the accumulation of solids in our system over many flush cycles, wherein blackwater is repeatedly disinfected and recycled, (2) the impact of repeated recycling on the energy requirements for effective disinfection and (3) efforts to improve energy efficiency of the electrochemical process under steady state conditions.

## Materials and methods

### Sample collection

Collection methods were approved by RTI's institutional review board. Urine and feces were collected from healthy volunteers 20–50 years of age using a portable urinal and 800‐cc graduated specimen collection pans, respectively, and stored at 4°C until use. Urine was stored for no more than 1 week; fecal samples were typically stored for 0–3 days and never more than 2 weeks.

### Prototype toilet liquid disinfection system

**Figure 1:**
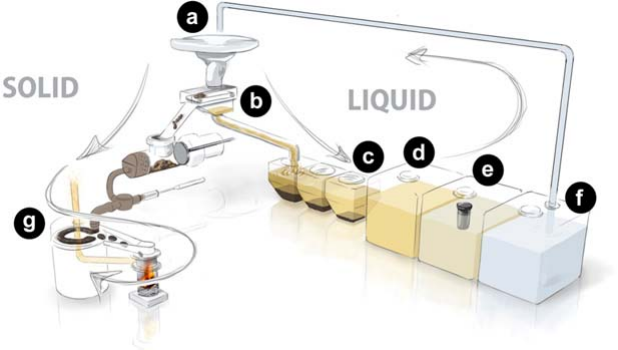
Prototype modular toilet system. (a) Squat plate user interface. (b) Solid–liquid separator. (c) Settling tanks. (d) Pre‐process holding tank. (e) Process tank. (f) Post‐process holding tank (reservoir for flush cistern). (g) Drying plate and combustor for processing solid waste. Figure reproduced with permission from Sellgren *et al*. (2017), RTI Press Publication No. RR‐0031–1704. Copyright 2017 RTI Press. [Colour figure can be viewed at wileyonlinelibrary.com]

The prototype toilet (Fig. 1) has been described previously (Sellgren *et al*. 2017) and includes a squat plate style toilet (Roca, 1.5 L per flush) connected to a custom‐fabricated solid–liquid separator. The solid–liquid separator houses a conveyer belt composed of 1/16″ × 1/8″ urethane bands spaced 1/16″ apart and effects efficient diversion (∼60–90%) of fecal solids from the liquid waste system to the solid waste system. The solid waste system and its testing will be described in detail in separate publications.

Liquid passing through the solid–liquid separator enters a settling system consisting of three, 12‐L polyethylene containers with 54° sloped bottoms, connected in series by plastic thermal welding via ∼1″ × 4″ openings cut near the tops of the tanks, yielding effective volumes of ∼10 L each. The final tank is connected via a gravity feed to a 60‐L polyethylene pre‐process holding tank. Liquid is pumped from the pre‐process tank through a 10‐μm filter into another 60‐L polyethylene tank (the process tank), containing a MMO electrochemical cell made up of 13 dual‐sided electrodes, 64 cm^2^ in area, separated by a 3‐mm gap (Hayward) and a paint stirrer used to agitate the liquid during treatment. Following treatment, the liquid is pumped through another 10‐μm filter to an additional 60‐L polyethylene holding tank, which serves as the supply reservoir for the flush cistern of the toilet.

### Flush cycles

Assumptions of toilet use patterns included two urinations and 1 defecation (with urination) per person per day, and an approximate 24‐h urine volume of 1.5–2.0 L per person (Rose *et al*. 2015). Two urine flush cycles were performed for each fecal flush cycle. Each urine flush contained 1.5 L of flush liquid (tap water for startup, treated recycled blackwater in subsequent cycles) and 0.5 L of urine. Each fecal flush contained approximately 150–200 g of wet feces, 1.5 L of flush liquid, an additional 0.5 L of flush liquid to simulate the use of an anal wash system and 0.5 L of urine. Thus, the process liquid requiring treatment contained ∼23% fresh urine in recycled flush water, contaminated with feces.

### Disinfection procedures and energy determination

For studies reported herein, waste water was treated in 30 or 60‐L batches. In pilot studies (data not shown), energy and time requirements for disinfection scaled linearly on a per volume basis up to 60 L (the maximum batch volume the system is designed to treat).

All disinfection runs were performed with continuous, vigorous agitation by a paint stirrer in the process tank (450 rpm) with care taken to ensure the electrode surfaces of the electrochemical cell were completely submerged. Power was applied to the electrochemical cell in constant voltage mode using a DC power supply. Voltage and current were recorded at regular intervals over the course of all runs. Electrochemical energy per volume of process liquid used at time *n* (*E_n_*) was estimated by:

(1) $$\;E_{n}=\;\frac{\;V\int_{0}^{n}I\left(t\right)dt}{v}$$

where *V* is the voltage, *I* is the current, *v* is the volume being treated. The integral of current with time was estimated by the trapezoid method.

### Water chemistry measurements

Oxidative reduction potential (ORP), pH and conductivity were measured using a Myron L 6PFCE Ultrameter II (Myron L Company, Carlsbad, CA, USA). The sensor wells were rinsed 3× with sample prior to measurement.

Free chlorine was measured using the *N*,*N*‐diethyl‐*p*‐phenylenediamine (DPD) method (HACH method 8167) with a HACH DR 900 colorimeter (HACH, Loveland, CO, USA) according to the manufacturer's instructions. Blanks consisting of sample effluent without DPD reagent were run prior to each sample to zero the instrument. Free chlorine pillow packs (HACH) were added to 10 ml of sample effluent, allowed to react for 1 min, then run using program 87 Chlorine F&T PP MR. Samples above the range of detection (2.2 mg/L) were diluted with diH_2_O, and the instrument was zeroed with the diluted sample prior to the addition of reagent. Exposure time (*C*·*t*) at time *n* was calculated by determining the area under the concentration/time curve (Le Dantec *et al*. 2002):

(2) $$\;C\cdot t_{n}=\left(C_{n-1}\cdot t_{n-1}\right)+[\frac{C_{n}+C_{n+1}}{2}]\cdot (t_{n+1}-t_{n})$$

Total solids (TS) were determined according to the EPA method (EPA 2001) by evaporating triplicate samples (5–10 ml) at 103–105°C in tared aluminum weighing dishes. Total suspended solids (TSS) were determined in triplicate by weighing liquid samples (∼10 ml) in tared tubes, washing the samples through tared 0.7‐μm filter paper (Fisher), and weighing the filter papers after drying at 103–105°C.

### Microbial enumeration and data analysis

Microbes were enumerated by the most probable number (MPN) method as previously described (Sellgren *et al*. 2017). Briefly, 5–10 ml samples of process liquid were drawn from the process tank during disinfection runs using sterile pipettes and placed in sterile centrifuge tubes, which were stored at 4°C until plating. Serial dilutions (10^−1^–10^−8^) of each sample were made in triplicate in lysogeny broth in sterile 48‐well cell culture plates. Samples were incubated at 37°C for 48 h before being analyzed.

Disinfection energy thresholds for MPN = 10^3^/ml and MPN = 5/ml for each disinfection run were interpolated from the plots of log (MPN) versus *E_n_*. In the cases where MPN values crossed those thresholds more than once, the threshold was defined as the first point at which the plot crossed that threshold and remained below it for all subsequent measurements. Statistical tests were performed as indicated using GraphPad Prism v. 7.01.

## Results and discussion

### Inputs to the system

The flush cycles performed for the characterization of steady state running conditions with recycled flush liquid are summarized in Supporting Information Table S1, and were performed on 25 non‐consecutive days over a 15‐week period. (Additional flushes were performed for the purposes of generating material for additional disinfection trials; the flushes presented in Supporting Information Table S1 are representative of all flush cycles performed for experiments presented in this report.)

There is considerable individual variation in fecal and urinary output; however, a recent comprehensive review puts the global medians at ∼128 g of feces (wet weight) and ∼1.6 L of urine per person per day (Rose *et al*. 2015). With these values and the flush rates used in this study (approximately 3 L recycled blackwater for every L of fresh urine), this comes to ∼20 g feces/L total liquid in the system; in the present study, our input was 22 ± 5 g/L (Supporting Information Table S1), suggesting that our process liquid in laboratory conditions mimics expected conditions in the field.

### Defining steady state in recycled process liquid

**Figure 2:**
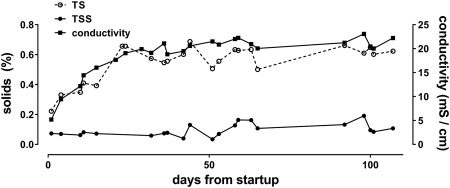
Accumulation of solids. Samples were taken from the process tank prior to electrochemical treatment. For TS and TSS measurements, numbers shown represent means of triplicate measurements.

Recycling blackwater resulted in a process liquid that was consistently alkaline, with low ORP, high turbidity (Supporting Information Table S2) and microbial counts (MPN) ranging from 10^5^ to 10^8^/ml, with most measurements on or near the order of 10^7^/ml (Supporting Information Fig. S1). Conductivity and TS rose rapidly through the first 6–7 flush cycle sets before achieving steady state ranges of ∼20–23 mS/cm and ∼0.50–0.65%, respectively, and remained stable over many subsequent cycles (Fig. 2). TSS ranged from 0.034 to 0.191%. The steady state conductivity was higher than that of the collected, pooled urine, which typically ranged from 13 to 16 mS/cm, suggesting the remainder of dissolved electrolytes found in the process liquid came from feces and/or from the degradation of organic constituents into more conductive species by resident microbiota in the pre‐process tanks and/or the electrochemical process.

### Recycling blackwater increases energy required for electrochemical disinfection

**Figure 3:**
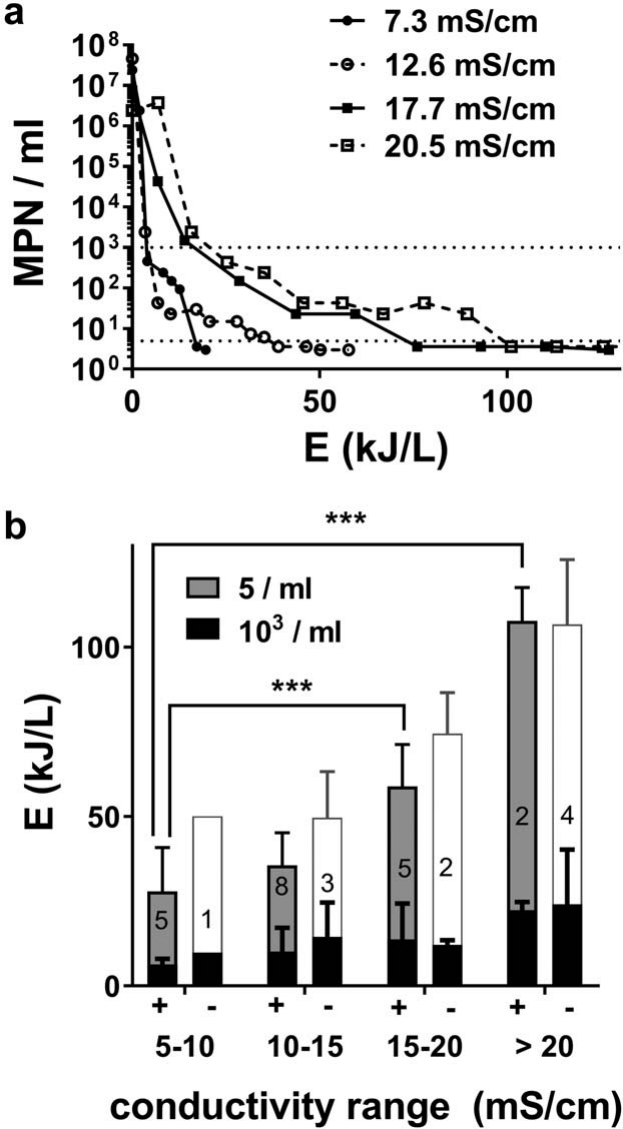
Impact of solids accumulation on disinfection energy. (a) Representative kill curves showing most probable number (MPN) over the course of EC treatment versus EC energy at different process liquid conductivities. Dotted lines indicate disinfection thresholds (10^3^ and 5 MPN/ml). (b) Disinfection trials (all conducted at in constant voltage mode at 24 V) were segmented based on conductivity of the process liquid at the beginning of the trial. “+” indicates trials in which complete disinfection (MPN < 5/ml) was achieved; “‐” indicates trials where this threshold was not achieved. Bars indicate the mean energy (±S.D.) required to achieve the indicated most probable number thresholds, with the number of trials per condition indicated by the numbers inside each stack of bars. The white bars in the “‐” trials indicate the highest EC energy recorded without reaching the disinfection threshold in those trials. Total energies required to achieve complete disinfection in the “+” trials were compared by one‐way ANOVA with a Dunnett's multiple comparison, test., *** = *P* < 0.001 for the comparisons indicated.

Initial disinfection runs were performed at 24 V as previously described (Sellgren *et al*. 2017). As conductivity (and TS) of the process liquid increased with repeated recycling, the energy required to achieve the desired threshold of disinfection (5 MPN/ml) also increased significantly (Fig. 3). However, the energy required to achieve significant levels of microbial deactivation (below 10^3^ MPN/ml) changed very little with increasing conductivity; rather, the increase in total energy demand could be almost completely accounted for by an increase in the energy required to deactivate the last 2–3 log units (Fig. 3b). Note that in some trials, the 5 MPN ml^−1^ threshold was not reached at all, indicating the average disinfection energies from the positive trials likely underestimate the energy required for full disinfection.

**Figure 4:**
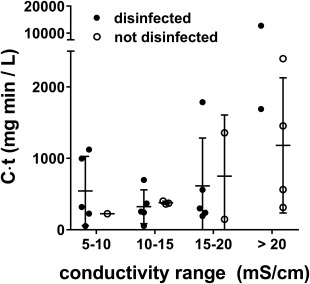
Free chlorine contact time does not predict complete disinfection. Shown are estimated *C*·*t* values for free chlorine in trials across different conductivity ranges (same trials shown in Fig. 3) at complete disinfection (MPN < 5/ml) or the highest value measured in trials where complete disinfection was not achieved.

The increase in energy required for full disinfection was accompanied by a trend towards increased *C*·*t* at the time of disinfection, but with considerable variability in *C*·*t* from trial to trial (Fig. 4). While most trials achieved complete disinfection at *C*·*t* < 1000 mg·min/L, a number of trials either failed to achieve complete disinfection at comparable contact times or required significantly greater *C*·*t* (up to 13 000 mg min/L) in order to achieve complete disinfection. Taken together, these data indicate that *C*·*t* is an unreliable parameter by which to predict complete disinfection of recycled blackwater, but also suggest that strategies to maximize *C*·*t* for a given electrochemical energy input may decrease the energy demand for disinfection.

### Effects of varying voltage and on/off time

**Figure 5:**
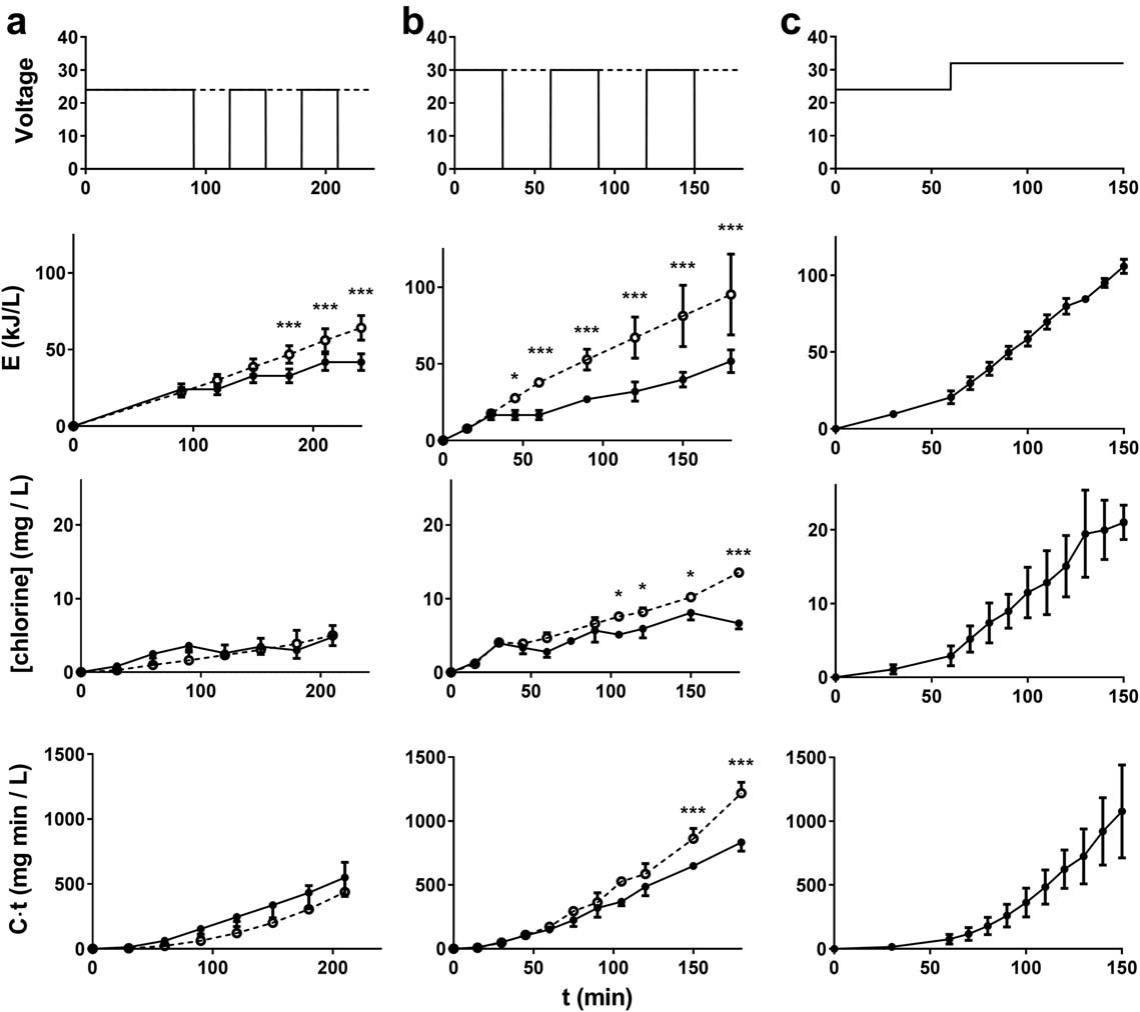
Summary of different operation cycles under steady state conditions. (a) Comparison of 24 V (open circles, dotted lines) and 24 V red (closed circles, solid lines) cycles, data from 6 to 4 independent runs, respectively. (b) Comparison of 30 V (open circles, dotted lines) and 30 V red (closed circles, solid lines), data from 2 to 3 independent runs, respectively. (c) Data from 6 independent runs with a 24 + 32 V cycle. (a and b) Results from continuous and reduced operation cycles were compared by two‐way ANOVA with a Sidak's multiple comparison test. * = *P* < 0.05, *** = *P* < 0.001. All panels: data are mean ± S.D.

In preliminary studies, we determined that when the electrochemical cell was powered off, the average estimated half‐life of free chlorine in our process liquid was 80 min (range 29–130 min) when MPN was > 5/ml, and 167 min (range 122–188 min) when below the disinfection threshold, comparable to reported half‐lives in treated water distribution systems (Olivieri *et al*. 1986); this is potentially long enough to increase contact times per energy used by operating the electrochemical cell in a pulsed on/off cycle rather than in continuous operation. To test this, we devised low‐voltage (24‐V) and high‐voltage (30‐V) cycles that pulsed on and off at 30 min intervals after a free chlorine concentration >2 mg/L was achieved, which, for 30‐L batches, was after 90 and 30 min of run time, respectively. The pulsed conditions were compared with continuous operation at the same voltages (Fig. 5a,b), in process liquid at steady state (Fig. 2), defined as having a conductivity >20 mS/cm.

In the 24‐V trials (Fig. 5a), pulsed operation was associated with decreased electrochemical energy for a given run time, as expected. When all disinfection runs were taken into account, there was no difference in free chlorine measured over time between the pulsed and continuous operating conditions, nor in the *C*·*t*. In the 30‐V runs (Fig. 5b), the difference in electrochemical energy was larger between pulsed and continuous conditions, and the free chlorine and *C*·*t* were significantly higher in the continuous condition at run times >90 and >150 min, respectively. However, at both voltages, pulsed operation led to a slightly, but consistently higher energy requirement to achieve the same *C*·*t* than continuous operation (Supporting Information Fig. S2).

**Figure 6:**
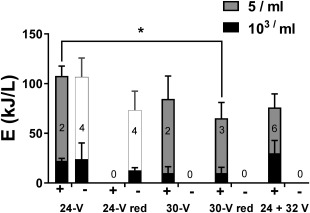
Efficacy and energy efficiency of different operation cycles. Disinfection trials conducted on “steady state” effluent (conductivity > 20 mS/cm) with different voltages operation cycles are shown. “+” indicates trials in which complete disinfection (MPN < 5 ml^−1^) was achieved; “‐” indicates trials where this threshold was not achieved. Bars indicate the mean energy (±S.D.) required to achieve the indicated most probable number thresholds, with the number of trials per condition indicated by the numbers inside each stack of bars. The white bars in the “‐” trials indicate the highest EC energy recorded without reaching the disinfection threshold in those trials. Note that the data shown for 24 V are the same as presented for “>20 mS/cm” in Fig. 3b, included here for reference. Total energies required to achieve complete disinfection in the “+” trials were compared by one‐way ANOVA with a Dunnett's multiple comparison, test. * = *P* < 0.05 for the comparison indicated.

Evaluation of the disinfection efficacy and energy efficiency of these different cycles is presented in Fig. 6. Complete disinfection was not achieved in any of the 24‐V reduced cycle (24‐V red) runs (longest run time was 600 min). However, both the 30 and 30‐V red cycles consistently achieved complete disinfection and with slightly improved energy efficiency over the 24‐V condition. Interestingly, the energy required to meet disinfection thresholds in all conditions was independent of starting MPN (Supporting Information Fig. S3).

While energy optimization is a high priority for the development of this technology, another priority is the ability to establish operating parameters that are easily programmed (such as run time), that do not require real‐time feedback from the system or operator intervention, and will reliably disinfect the process liquid every time. Though the disinfection thresholds were consistently achieved within a narrow range of electrochemical energy expenditures for each operating cycle tested, variability in the conductivity and current across individual runs meant that actual run times varied widely (e.g. 30‐V disinfection times ranged from 105 to 240 min, Supporting Information Fig. S4) and required continuous user monitoring to determine how long the disinfection run needed to be performed.

With the goal of establishing run times and voltages that will meet the criteria necessary for independent/automated operation, an additional operational cycle was tested consisting of a 24‐V phase for 60 min followed by a 32‐V phase for 90 min (Fig. 5c). This cycle was chosen based on the observations that: (1) 24 V was sufficient for deactivation of microbes down to 10^3^/ml (Fig. 3b) and with comparatively low free chlorine levels (Fig. 5a), (2) raising the voltage reduced overall run time required to achieve complete disinfection and (3) at steady state process liquid conditions, higher voltage more consistently achieved complete disinfection (Fig. 6). Thus, the combined voltage cycle represented a compromise between the goals of maximizing energy efficiency and establishing reliably efficacious run times. Across six trials, this operational cycle achieved the threshold for complete disinfection every time, with energy efficiency matching the 30‐V runs (Fig. 6), but with a shorter and more predictable run time (<150 min total for all runs, Supporting Information Fig. S4) than any other cycle tested.

With the 24 + 32 V operating cycle, the average energy expenditure for the electrochemical process to achieve complete disinfection was 76 kJ/L (21 Wh/L.) On a per volume basis, this is approximately 10 times the energy requirement recently reported (2 Wh/L) for a similar electrochemical process to achieve a 5‐log reduction in model bacteria added to dilute toilet waste water (Huang *et al*. 2016). Our system has been tested exclusively with concentrated blackwater representing anticipated field conditions (including a diverse population of microorgansims), with starting microbial counts typically on the order of 10^7^ ml^−1^ and with an absolute (rather than relative reduction) threshold for complete disinfection, which is essential for recycling. Given the “long tail” shape of our disinfection curves (e.g. Fig. 3a) which indicate that the majority of the electrochemical energy is spent on deactivating the last 1–2 log units, we postulate that the disparity in energy efficiencies between the systems can be accounted for by the different test media and evaluation criteria.

While the present findings will guide the next phase of prototype tests in the field, future laboratory‐based studies will address what characteristics of recycled blackwater drive its comparatively high disinfection energy demand and the long tail phenomenon, including chemical oxygen demand (Lopez‐Galvez *et al*. 2012) and association of microorganisms with particles (Berman *et al*. 1988, Emerick *et al*. 1999), and how best to remediate them in order to achieve more energy efficient disinfection.

## Conclusions

1. Repeated recycling of disinfected blackwater in a free‐standing waste water treatment application leads to accumulation of solids and electrolytes in the process liquid, which rapidly achieve a steady state.
2. Accumulation of solids is associated with significantly increased energy requirement to achieve complete disinfection with an electrochemical process. Thus, a clear definition of steady state is essential to making meaningful comparisons of different operating parameters in this system.
3. Raising the operating voltage to 30 or 32 V modestly decreased the energy required for complete disinfection of steady state effluent compared to 24 V; a two‐stage process with a 24‐V phase followed by a 32‐V phase matched this efficiency with a more reliable run time.
4. These findings are critical to moving this technology from the laboratory setting into the field, where the system will need to operate without the benefit of continuous monitoring and under conditions in which the inputs are not readily controlled.

## Supporting information

Additional supporting information may be found in the online version of this article at the publisher's web‐site

Supporting Tables and Figures
