# Supplementary material for: Electrochemical disinfection of repeatedly recycled blackwater in a free‐standing, additive‐free toilet
Source: Water Environ J. 2017 Jul 23;31(4):545–51. doi: 10.1111/wej.12277 (PMC5724497; doi:10.1111/wej.12277)
Supplement: Supplementary file 1 — Supporting Tables and Figures [file WEJ-31-545-s001.docx]

| **date** | **V _urine_ (L)** | **V _flush liquid_ (L)** | **V _total liquid_ (L)** | **m _feces_ (g) (mean ± S.D., n)** | **m _feces_ (g) / L** |
| --- | --- | --- | --- | --- | --- |
| Oct 13 | 8.5 | 28.0 | 36.5 | 216 ± 57, 5 | 30 |
| Oct 15 | 7.0 | 23.0 | 30.0 | 137 ± 26, 4 | 18 |
| Oct 20 | 8.5 | 28.0 | 36.5 | 180 ± 103, 4 | 25 |
| Oct 22 | 8.5 | 28.0 | 36.5 | 182 ± 77, 5 | 25 |
| Oct 26 | 5.5 | 18.0 | 23.5 | 201 ± 80, 3 | 26 |
| Nov 3 | 8.5 | 28.0 | 36.5 | 190 ± 55, 5 | 26 |
| Nov 5 | 7.0 | 23.0 | 30.0 | 153 ± 66, 4 | 20 |
| Nov 9 | 7.0 | 23.0 | 30.0 | 117 ± 25, 4 | 16 |
| Nov 11 | 7.0 | 23.0 | 30.0 | 113 ± 34, 4 | 15 |
| Nov 16 | 7.0 | 23.0 | 30.0 | 145 ± 95, 4 | 19 |
| Nov 17 | 8.5 | 28.0 | 36.5 | 150 ± 73, 5 | 20 |
| Nov 20 | 8.5 | 28.0 | 36.5 | 162 ± 98, 5 | 22 |
| Nov 24 | 7.0 | 23.0 | 30.0 | 120 ± 35, 4 | 16 |
| Nov 30 | 8.5 | 28.0 | 36.5 | 193 ± 66, 5 | 26 |
| Dec 3 | 7.0 | 23.0 | 30.0 | 128 ± 46, 4 | 17 |
| Dec 4 | 7.0 | 23.0 | 30.0 | 118 ± 60, 4 | 16 |
| Dec 8 | 6.5 | 21.5 | 28.0 | 198 ± 105, 4 | 28 |
| Dec 11 | 4.0 | 13.0 | 17.0 | 187 ± 17, 2 | 22 |
| Dec 15 | 7.0 | 23.0 | 30.0 | 162 ± 39, 4 | 22 |
| Jan 8 | 7.0 | 23.0 | 30.0 | 218 ± 33, 4 | 29 |
| Jan 12 | 5.5 | 18.0 | 23.5 | 231 ± 145, 3 | 29 |
| Jan 14 | 7.0 | 23.0 | 30.0 | 203 ± 74, 4 | 27 |
| Jan 19 | 7.0 | 23.0 | 30.0 | 143 ± 95, 4 | 19 |
| Jan 20 | 7.0 | 23.0 | 30.0 | 142 ± 53, 4 | 19 |
| Jan 26 | 7.0 | 23.0 | 30.0 | 142 ± 53, 4 | 21 |
|  |  |  |  |  |  |
| **Table S1:** Summary of flush cycles performed | | | | | |

|  |  |  |
| --- | --- | --- |
| **parameter** | **mean ± S.D.** | **range** |
| **pH** | 8.76 ± 0.12 | 8.16 - 8.88 |
| **ORP (mV)** | -223 ± 121 | -432 - +5 |
| **turbidity (NTU)** | 760 ± 195 | 282 - 1000 |

**Table S2:** Pre-treatment characteristics of blackwater process liquid

**
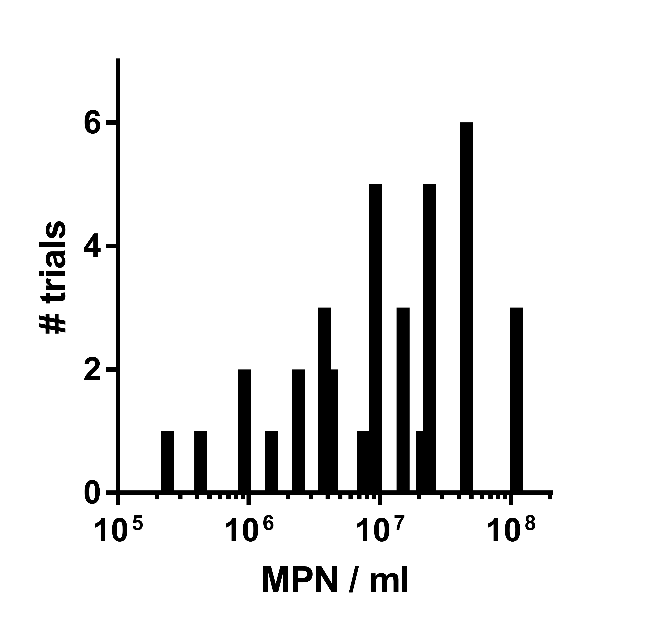
**

**Figure S1:** Frequency distribution of pre-treatment MPN in blackwater process liquid over 36 trials


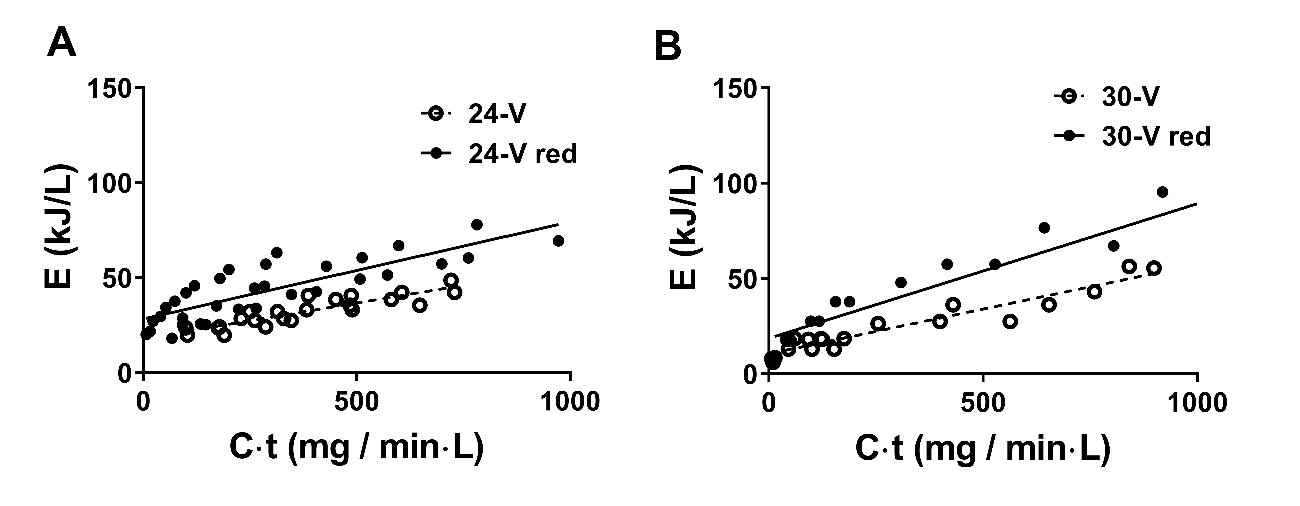


**Figure S2:** Electrochemical energy expended to achieve a given C·t at 24 V (A) and 30 V (B). Data are the individual points summarized as mean ± S.D. in Figure 5A and B, presented here as scatter plots. Lines indicate the linear regressions of each data set.


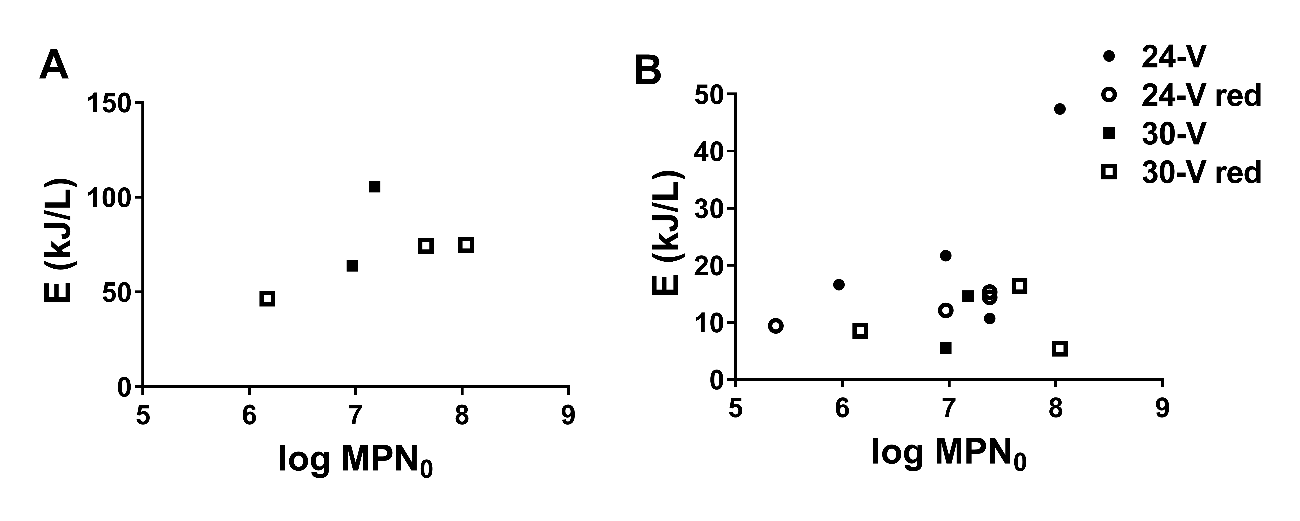


**Figure S3:** Energy required to reduce MPN to 5 ml^-1^ (**A**) and 10^3^ ml^-1^ (**B**) is not dependent on initial MPN (MPN_0_). Data are from the individual disinfection trials shown in Figures 5 and 6.

**
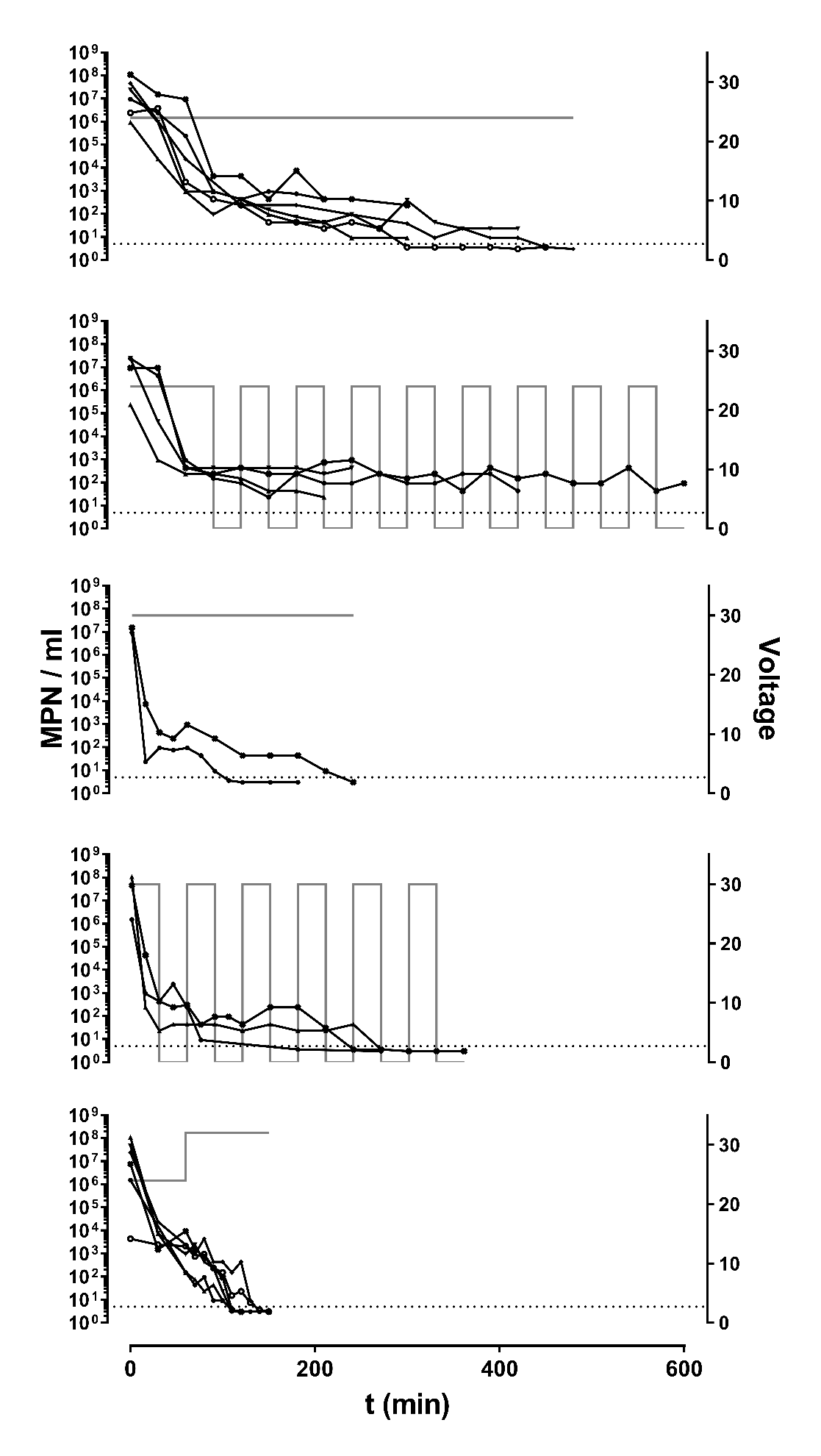
**

**FigureS4:** Kill curves with respect to run time for all runs summarized in in Figures 5 and 6. MPN are in black (left axis), operating voltages are in grey (right axis). Dotted line = threshold for complete disinfection (MPN 5 / ml).
